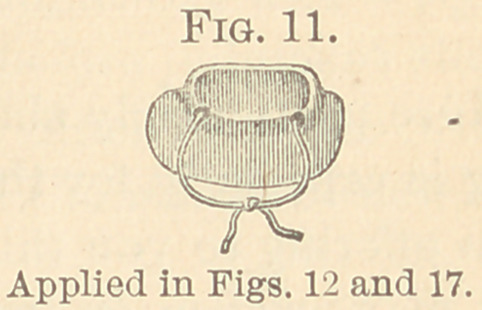# Regulators and Methods of Correcting Irregularities

**Published:** 1889-01

**Authors:** W. G. A. Bonwill

**Affiliations:** Philadelphia


### REGULATORS AND METHODS OF CORRECTING IRREGULARITIES.*

BY W. G. A. BONWILL, D.D.S., PHILADELPHIA.

My first essay on orthodontia was written in 1862. To make my own history more replete, however, it is necessary to show what I have done in this line of work since 1854. As the apparatus was then entirely new and the practice considered rather radical for the time, and as it has since been revived by others, I shall briefly present them here.

From the following language it will be seen that the "Coffin plate" of rubber was anticipated by me, except that I used silver wire made spiral, and adjustable or detachable from the plate previous to 1862

"If the inferior jaw, I clasp, where possible, and when not, *strike* up a plate to cover the deciduous or permanent teeth, as they may be, and operate from this. From the inward inclination of the inferior bicuspids and molars (or molars alone of the temporary set) there will be sufficient firmness gained by making it to press outward at these points."

"If there are no other means of holding it in the inferior jaw, an india-rubber plate made to fit accurately either the teeth or palate, or both; and if you desire, the surface of the vulcanized plate can be roughened to enable the patient to masticate thereon, and screw the spiral springs into this."†

This I seldom use, being bulky and dirty, and far more liable to injure the faces of the teeth. More can be done with the spiral spring soldered to a metal plate.

The same principles of action I still adhere to, namely:

1st. To commence as soon as possible after the seventh year, or as soon as there is evidence of decided irregularity.

2d. To watch all children's teeth from the third year and determine by an exploring needle, every three months, the exact position of the coming permanent teeth as soon as the first permanent molar has appeared.

3d. To preserve, by early treatment, the first and second molars—temporary—even to the treatment of their pulps, if the little patients are not brought in time to obviate it.

---

*Advance sheets from Harris's Principles and Practice of Dentistry, 12th edition.

†Extract from essay on Orthodontia read before the Delaware Dental Society in 1863, and which, on account of its length, was refused publication in the *Cosmos*.

4th. To be sure the first permanent molars are preserved without loss of pulp, and to allow nothing to interfere with their full and free development in the arches, as upon these teeth more than upon any others are due the irregularity, *from coming too far forward in the arch*, *from decay of approximal surfaces of temporary molars*, *or from the tardy eruption of the permanent incisors.* The sixth year molar drives the arch into smaller space when the incisors have appeared out of or inside the arch.

If the arch is once interfered with the area is not so great, and consequently there will be a deeper underbite, and the permanent molars will move forward and always keep it so, thus causing the permanent teeth, which are yet undeveloped in the maxilla, and lying over each other and not in line, to roll over and shorten the whole maxilla in front of the permanent molar. I, therefore, endeavor to keep this tooth as far back towards the ramus as possible.

5th. That all apparatus should be simple and, if possible, firmly fixed, so that the patient can have no control over it; and then see the case every few days.

6th. That constant and uninterrupted pressure is preferable. The antagonism of the opposite jaw will always be exerting a force to make them move back and forth in the sockets, and this makes sufficient intermittent pressure.

7th. That while one plan, without some change in each case, will not do, yet the infinite number of apparatus is a greater nuisance to patient and operator.

8th. That impressions of both jaws in plaster and a duplicate from the first; so that the plaster teeth can be cut off and rearranged to see the effect, and these models placed in the anatomical articulator, where they can be studied in the lateral movements, so necessary. That this shall be studied carefully; and, before action is taken, have the patient call and study the case in relation with the plaster model; and if doubt exists as to the extraction of a tooth or teeth, better postpone a few days and send for patient again rather than make so great a blunder.

9th. That a tooth shall be held as sacred as an eye; and, while extraction is sometimes demanded, when the greater good of the patient is at stake—when of weak constitution—yet do not too hastily resort to it.

10th. That without the combined assistance of parent and child better not commence.

11th. That nothing shall be withheld from the child or parent, but every detail, every risk, and the amount of patient endurance needed, the long time, and, when all is corrected, to allow of stay plates, that the work gained may be retained.

12th. Not least of all the factors, you must place such valuation on your services as will insure your interest and will drive the parties concerned up to their duties.

To these points I would now further insist on the great importance of utilizing as factors or fulcrums the temporary molars.

1st. By shaping them with a disk on all their sides or surfaces, so that a gold clasp can be securely placed thereon. Figs. 22 and 23.

2d. Where only a ligature is needed, to cut a groove with the disk on the buccal and palatal and lingual surfaces near the cervix, in which to place the silk ligature to keep it from working down under the gum. Fig. 22, C. C.

*These teeth will soon be lost, and no injury is done by shaping and grooving them.*

3d. By the use of gutta-percha (Figs. 217 and 218), warmed and placed on the palatal or lingual side of the tooth, around which a ligature is to be placed and carried slightly up over the grinding surface to prevent the ligature from pressing down under the gum. This I use on permanent teeth.

4th. Where the tooth cannot be cut or gutta-percha used, then gum sandarach varnish or a thin solution of oxyphosphate zinc placed on the tooth will prevent the ligature from slipping when the tooth is being rotated, or to keep it from pressing up under the gum.

5th. The immense importance of the Anatomical Articulator, with the geometrical and mechanical laws governing it.

The study of this alone will lead to the anticipation of so many irregularities, and will teach one to begin very early. It shows how invariable is law; and, when violated, where the cause is and how to obviate it.

It shows what is an *archetype*, and demonstrates clearly how the highest efficiency is reached in the equilateral triangular jaw of man, and that nothing can be made more perfect either by nature or by man.

To make understandingly the application of these laws to orthodontia much must need be unravelled that can be seen in the *American System of Dentistry*, vol. ii, page 486. Presuming upon your having read the article, advantage of this will be taken and time saved by putting the matter concisely.

What we want to get at is the insignificance of the grinding surfaces of the bicuspids and the molars with the curve at the ramus and the particular angle formed by the palatal surfaces of the superior incisors.

We want to know exactly how much the superior incisors should overlap the inferior, and how far up on the palatal surfaces of the superior incisors and cuspids the inferior should go—or the underbite—before we can understand what is a deviation, from the normal standard, and how to intelligently correct it.

I have asserted that the length of cusps of the first superior bicuspid governs the whole thing. Given such a tooth from a prehistoric age even, and it can be told how deep was the underbite of the inferior incisors. It is no guesswork.

You see hundreds of mouths with the inferior incisors going so far up that they touch the base of the superior incisors and in many cases the gums. Why is it not normal? No one can tell without knowing this law of the first superior bicuspid.

Every one assumes that the upper should close over the lower incisor; but gives as the only reason for it that the inferior being smaller in width must form a smaller arch, and must work within the superior arch of larger incisors.

There are several other reasons that this model will reveal, and could first be known and seen in it only.

Unless there is an underbite, and that regulated to a given depth by the teeth in the rear of the arch, the superior will be thrown too far forward, while the inferior will be thrown inward, so as to lose their usefulness and be a deformity, as we so often see when the bicuspids and molars are gone and no antagonizing surfaces are left as abutments. When we look at the curvature at the ramus we are reminded that there is an overbite, for were there no curve just here the muscles would act more forcibly on the side opposite to that upon which one is chewing, and the normal relation of compensation and efficiency would be destroyed.

Then I say the highest efficiency cannot be reached, or in other words, one cannot get the greatest results from the least expenditure of force, and with least wear to the teeth, except by following this design. When this is fully realized, you will see where but little change of position of the *first permanent molar* forward, from the extraction of a temporary molar, the normal bite is made much deeper, as it then allows the jaws to approach each other very much faster.

If nature intended to have given man a deep underbite, then we should see such an arrangement of the back teeth such as carnivorous animals have, where but one long cusp is used to get the greatest amount of shearing surface.

Instead, then, of the bicuspids having cusps greater than the angle of an equilateral triangle, they would all be cuspid teeth in order that the cusps might be of value in the lateral movements. When they reach beyond an angle of 45°, efficiency is no longer gained; but a direct loss and danger of fracture by the long wedge-shaped cusps that would have to enter it. A cuspid would be much more powerful to pierce and cut, and no danger of loss from fracture.

Then I assert, when the overbite or rather underbite rises higher than *one-eighth of an inch*, abnormality begins; since the incisors will not permit the bicuspids and the molars to come into contact when the incisors are touching on their edges. But, by this arrangement, no matter in what lateral position you place the lower jaw, the teeth of both jaws will be touching at their separate points of the equilateral triangle at once.

We will take a natural superior first bicuspid and measure the length of its cusps, place it at the point on the two lines *a* and *e* in its relation in distance from the condyles with the superior centrals.

FIG. 1.

This will be about one-fourth the distance from the centrals to the line running from the condyles. You will now see that if these two lines *a* and *e* diverge from the point of motion at the condyles at T until they reach the superior bicuspid at *b* at the depth of groove, that by carrying the lines still further to the left until they strike the palatal surface of the superior incisor, the lines must be further apart than anywhere else. Fig. 1.

By this one knows exactly, when grinding on artificial teeth, that if the overbite at *e* and *a* is one-eighth of an inch, the depth of cusps of all the teeth backward until T is reached would be of less depth, and at T would have no cusps at all.

Were this not true, only certain teeth would touch at any lateral movement; and deeper than this, the bicuspids and molars, touching but little of the time, would throw much more force on the palatal surfaces of the upper incisors to press them out of the arch, and contract the arch of inferior incisors and crowd them into a lesser arch, as so many bricks, one over the other.

It is thus apparent that the permanent molars must be in such a position that their length out of the jaw is such as to allow the inferior incisors to occupy a larger arch, and that only under such a plan can they be regular and fill their highest function.

To make the application. If we extract the first temporary molar too soon after the sixth year, the second temporary molar will be thrown forward on this scale and on these lines *a*, *e* to T, so as to allow the jaws to come closer together and force the inferior incisors further in under the wedge-shaped palatal surfaces of the superior incisors, until they begin to overlap one another; since the arch becomes less as they are driven backward by the inclined plane of the palatal surfaces of the superior incisors, and until the first molars again touch on their grinding surfaces, which is only after they have been moved forward between these lines *a* and *e*.

The same result follows should the first permanent molars not come up in the inferior jaw as fast as do the incisors. The latter are in advance, and consequently there is no prop long enough to hold the jaws from a deep underbite, or to prevent the inferior incisors from touching the gum.

Besides, if the inferior permanent incisors should be forced within the arch by non-absorption of the roots of the temporary teeth, they would have no guide from the superior incisors, and the result would be too deep an underbite.

Now this all occurs with the temporary teeth, and the first permanent molars at the seventh year.

Should the second temporary molar be extracted too soon, the deformity becomes more marked by the forward movement of the first permanent molar.

Aside from direct loss by extraction, much approximal surface is lost on all the temporary molars and on the incisors from caries. This allows the first permanent molar to move forward, and by this change in position the jaws fail to be kept apart, which, at this early age, is so necessary in order to anticipate the crowding of the arch. Still further is this condition increased by the rapid decay of the first permanent molars, allowing the jaws to approximate still nearer, forcing the lower incisors into a much smaller arch, and consequently higher up under the superior permanent incisors.

Add to all this the crowning climax of blunders: the extraction of the first permanent molar or molars too soon. One is enough to break up the masticating surface on that side, and how great is the loss, since the force of mastication is thrown upon the incisors, which, at this early age, must drive the upper out and the lower in, and thus cause them to crowd worse than ever.

Let us now observe the condiiton of the permanent teeth in the jaws, not yet to the surface (bicuspids and cuspids), with a contracted arch, from the full complement of teeth, but with the loss of mastication in the proper region, which has prevented the expansion of the arches that is so necessary at this early age.

Even if none are extracted, the many deciduous teeth give untold pain from exposure of abscesses preventing the use of the jaws on hard food, such as is needed to develop size and bring more nutrition to the parts to make the processes.

Is it at all wonderful that we have increasing abnormality with increase of caries?

Can we not see from this pen picture what a grand field we have for shaping the destiny of individuals who are certainly doomed to greater deformity as the ages come?

Your duty lies, above all else, in watching each child with scrupulous care, making it a part of a *forced* education to go to the dentist every three months and submit to a close examination with an exploring needle to find the coming tooth in advance of looseness and also to give your best efforts to the saving of the temporary teeth before the pulps are exposed.

The principle causes of irregularity are diverted and "polluted" nutrition. Nutrition is *diverted* when the jaws and teeth are not actively and normally used, and it is taken up by those organs that are demanding it from their constant action before it can be applied to the bones. *Polluted* nutrition is where diseases of various kinds contaminate the fluids and render inactive by their poisons the organs so as to cause irregular deposits of bone in the teeth and maxillæ, retarding their growth, and consequently their arrangement, in the arch.

The choice of proper food and its mastication has a powerful effect, not only in diminishing the supply of phosphates, but also their application to the jaws and teeth from want of proper action.

The trigeminus nerve, to my mind, is not a factor.

If, then, nutrition by perversion, pollution, or diversion is a prime factor in the cause of irregularities, let us imagine the first effort that nature makes to supplant the temporary set. The first permanent molar in both jaws should be present and to their full height or place, or in contact before the central incisors are lost.

Early decay of the temporary teeth is potent in irregularities. So also is the injudicious use of the forceps, by the early or late extraction, accidents, and, not least, the meddlesome dentist. We find it almost entirely confined to civilized life. It is never found in the lower animals.

The muscles are becoming, or should be, stronger every day, as most active parts gain the most nutrition and at once. Unless the teeth are in full contact and well propped in position by alveolar borders calculated to resist the coming force to be exerted thereon, the arches in front being now the weakest of all, must be pressed out of position by the jaws being forced nearer each other. This easily occurs if nutrition has not been plentiful and has not been eagerly used in the formation of the alveolar processes.

Consider the average set of teeth of the child of six that comes to us. The further the sixth year molar goes forward between these two lines *a*, *e*, and T, the less room all the coming permanent teeth have; and, the jaws necessarily coming closer together than if the molars had remained at their place in the alveolar border, where the greatest resistance is offered, we can see how the bicuspids are rolling over one another; and in the circle, or arch, in front where the teeth are in advance in the lower jaw, but not growing as fast as they should from want of nutrition by perversion or pollution, the props—the temporary incisors—being no longer of value, the jaws approximate too closely; so when the laterals make their appearance, is it any surprise to us that they should be inside the arch in most cases? How could they arrange themselves regularly when the arch is so wanting in bone firm enough to hold them in bounds, and is suffering from caries of all the posterior teeth, while those not yet above the surface are crowding forward as the resistance is taken away owing to the condition of the temporary molars and to the lack of energy in the tissues, both soft and bony? It would be marvellous if they, in their normal state, which is apparently not in curve, should not be found one upon the other, overlapping in the border before eruption.

To add to the trouble, the irregularity of their periodicity is so great and out of proportion, that the inferior permanent incisors are crowded into a smaller arch than the third of a circle, and there is not room for them. The malady increases as the incisors of both jaws come into place. If there is any irregularity in the superior jaw, it then becomes greater in the lower; for as the inferior teeth reach the normal point, where they should stop going up under the superior on the palatal side, they fail to do so for want of that proper resistance which a perfect arch alone in the lower would insure.

But one tooth inside the arch of the lower jaw and at once, as the superior come into place later from requiring more nutrition and want of full use from the pain of mastication, they are retarded and the lower arch becomes smaller than the third of the circle; and, as a sequence, they are crowded by the superior inward and upward until in so many instances they reach the gums at the base of the superior incisors, because, as I have said previously, the lower arch had collapsed from the many causes which should have been prevented.

This may occur in jaws where the temporary teeth are in perfect condition, as well as the sixth year molars, from a contracted alveolar border, and from absorption's not keeping time with the advance of the teeth. But it is not so often found.

Instead of *contracted jaws from extraction and caries, it is* the *compressed alveolar borders*, and the want of resistance in them, which prevents normal mandibular action, and consequently healthy nutrition cannot result.

Keep back the first permanent molars, and if possible, push them further back towards the condyles, that there may be no intrusion on the domain of the coming permanent teeth. Have the temporary teeth in such condition that free mastication can be performed. Give the child all the nutritious food it needs, with plenty of exercise and sleep. Keep saccharine matter, in the shape of cake and candy. far from it. Make it eat its food without liquids. Have the salivary glands of value by compelling them to secrete from the use of dry food, and food will be sweet enough without sugar. The food is kept longer in the mouth. The jaws are used their full time. The muscles become stronger and the alveolar borders firmer, and the nutrition is plentiful, and is utilized without any part having to cry out for want of it. The nutritious supply will then always be in advance of the demand, and well laden with everything that can give life to the organs; and good teeth and a well arranged mouth will result. Let us now consider methods of regulating.

The figures from 2 to 23 show all my appliances and their applications for irregularities. Figs. 2 to 7 show the spiral spring in various phases and which are illustrations of the original apparatus for the correction of irregularities used in illustrating my paper read before the Delaware Dental Society in 1863, above referred to, page 10. It will be observed that the "Talbot spiral spring" is a true reproduction of the figures 2 to 7. These I used for several years; but have now almost abandoned for the present simple device, shown in Figs. 8 to 11.

Fig. 2.

Fig. 3.

Fig. 2 represents a silver plate made to fit the inferior incisors, and which was tied on a central, to correct a superior central from the inclined projection on the right; the end of spring acted on the right inferior central to throw it out of the arch.

Fig. 4.

Fig. 5.

Figs. 3, 4, 5 represent metal bands with clasps, with the spiral spring soft-soldered under a metal loop hard-soldered to the band. This retains the temper. These are used on many teeth in either jaw.

Fig. 5 shows a metal plate with half-clasps fitted to the bicuspids, to hold it in position. The spiral spring is soft-soldered to the plate. This can be changed to various positions on the plate, and is applicable in cases where it is difficult to place the clasp entirely around a tooth.

Fig. 6 was made for drawing backward the four incisors of the inferior jaw with spiral springs, adjusted so as not to interfere with the tongue or the superior teeth. The piece at A goes over the incisors, and is held by ligatures tied to one or more of the teeth.

FIG. 6.

FIG. 7.

Fig. 7 shows a jack-spring for constant pressure. It may be made in a curve to conform to the hard palate. It is very powerful and effective, and superior to a *jack-screw.*

In all these spiral-spring appliances, the spring is tied to the tooth to be acted upon to hold it from slipping; or, in some cases, a hole drilled into the tooth is better.

The appliances that with me have superseded all others are seen in Figs. 8 to 23. Fig. 8 represents a curved bar made of platinized gold with four holes punched for the passage of silk ligatures.

FIG. 8.

Applied in Fig. 16.

FIG. 9.

Applied in Fig. 20.

It is another way of applying Fig. 9 without band, and is used mostly for a single tooth in either jaw. The principle of action will be seen in Fig. 16, where two inferior lateral incisors are to be drawn from within out. To do so requires expansion of the jaws. This is effected by making the holes in the end of the plate over the centre of each cuspid, and by carrying the silk ligature from the mesial side of the laterals around back and up between the lateral and the cuspid and through the hole in the plate at either end, and attaching to it a rubber band which is stretched between the holes. This pushes the cuspids backward or opens the arch, and the centrals moving forward somewhat, the laterals easily fill the breach. Once in position they are retained without apparatus.

If the holes through which the ligatures pass were made exactly opposite the laterals, no good would be effected, because the pressure would be as much down as out, thus compressing the arch. But the ligatures applied as directed force the teeth apart, although the band is resting hard on the cuspids. The ligature is a loop or slipknot, and must be applied so as to come out between the lateral and the cuspid. Gum sandarach varnish will keep it

FIG. 10.

Bar shown as applie Fig. 12.

FIG. 11.

Applied in Figs. 12 and 17.

from slipping around the tooth. The band as heretofore applied has not expanded the arches, because the holes were not in the right places—over the cuspids.

Fig. 9 is this same bar with a clasp on one side of the arch. The bar is lengthened beyond the clasp to allow of the rubber tubing, tied at B, being attached far enough away from A in order to give suffibient power to move the teeth desired.

It was applied, Fig. 20, by clasping a first molar where the right central had to be twisted, and the lateral also, but in opposite directions. The bar rests upon the mesial buccal edge of the lateral while the silk ligature is carried *twice* around the central, bringing it up next the lateral, and over it through the hole in the bar at the point where it rests on the lateral, and is now drawn through the rubber band which has been tied opposite the molar. The rubber is stretched to the full length of the bar. The cuspid was also drawn outward on the same bar by boring a hole directly opposite, which was made to twist the cuspid as well as to draw it outward.

Fig. 10 is the same bar applied to Fig. 12 for drawing out both superior laterals and expanding the arch. The right cuspid was just emerging and the first bicuspid was clasped. The ligature with a slip-loop was carried over the right lateral, coming up from its distal side and through the hole in the bar at A, and tied to the rubber band near the first bicuspid. The left lateral was ligated the same way, coming up through the hole at B, which is over the center of cuspid. The ligature pressing the left cuspid backward was tied to the rubber band at C. Where the bar is too short to stretch the rubber band, it can be lengthened on one side of the clasp or carried back to the right bicuspid.

(*To be continued.*)

## Figures and Tables

**Fig. 1. f1:**
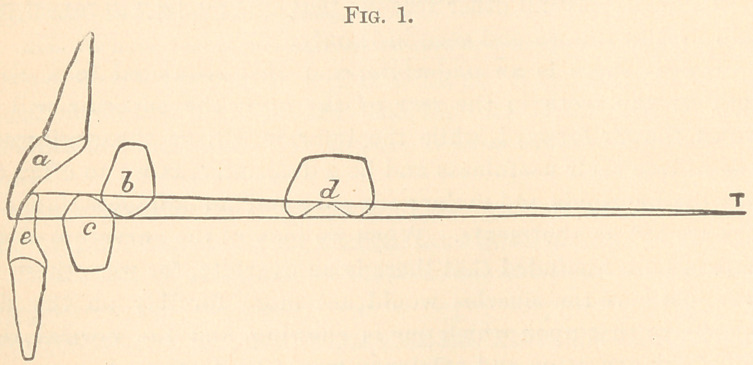


**Fig. 2. f2:**
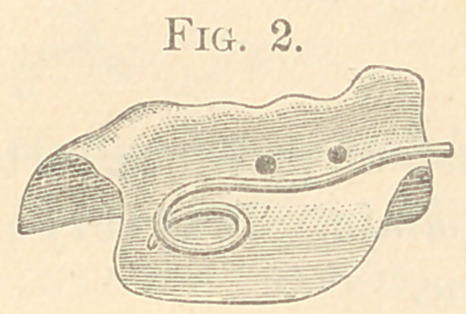


**Fig. 3. f3:**
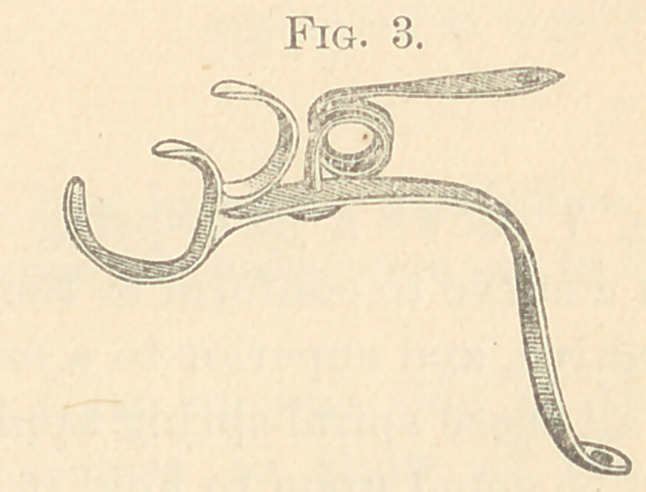


**Fig. 4. f4:**
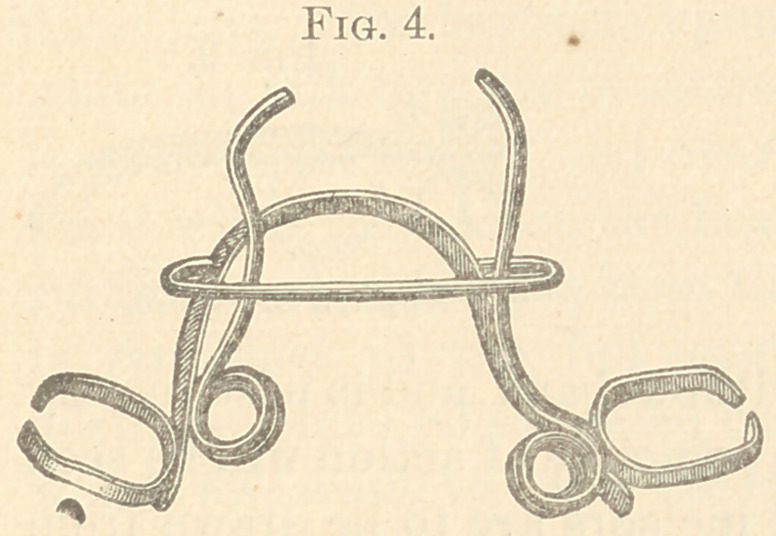


**Fig. 5. f5:**
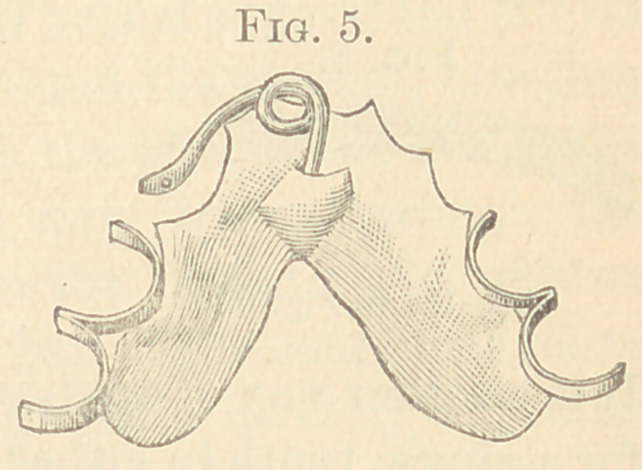


**Fig. 6. f6:**
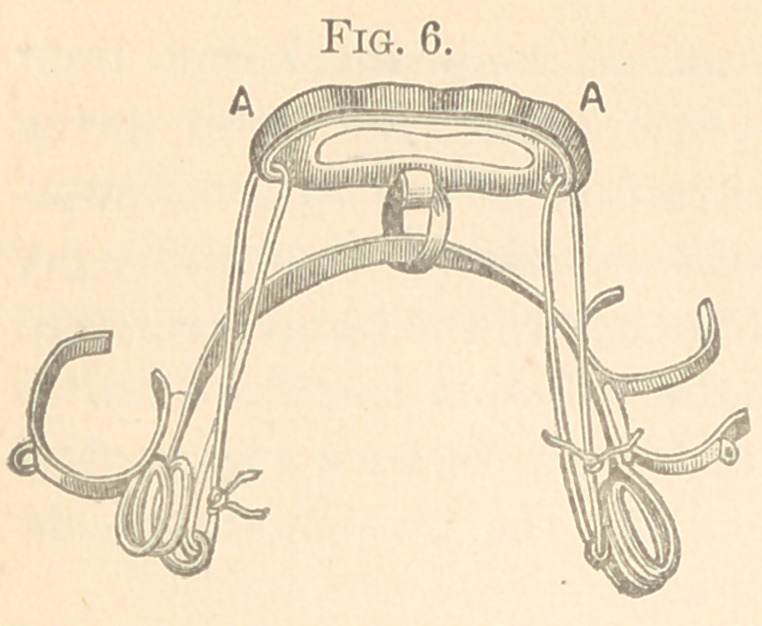


**Fig. 7. f7:**
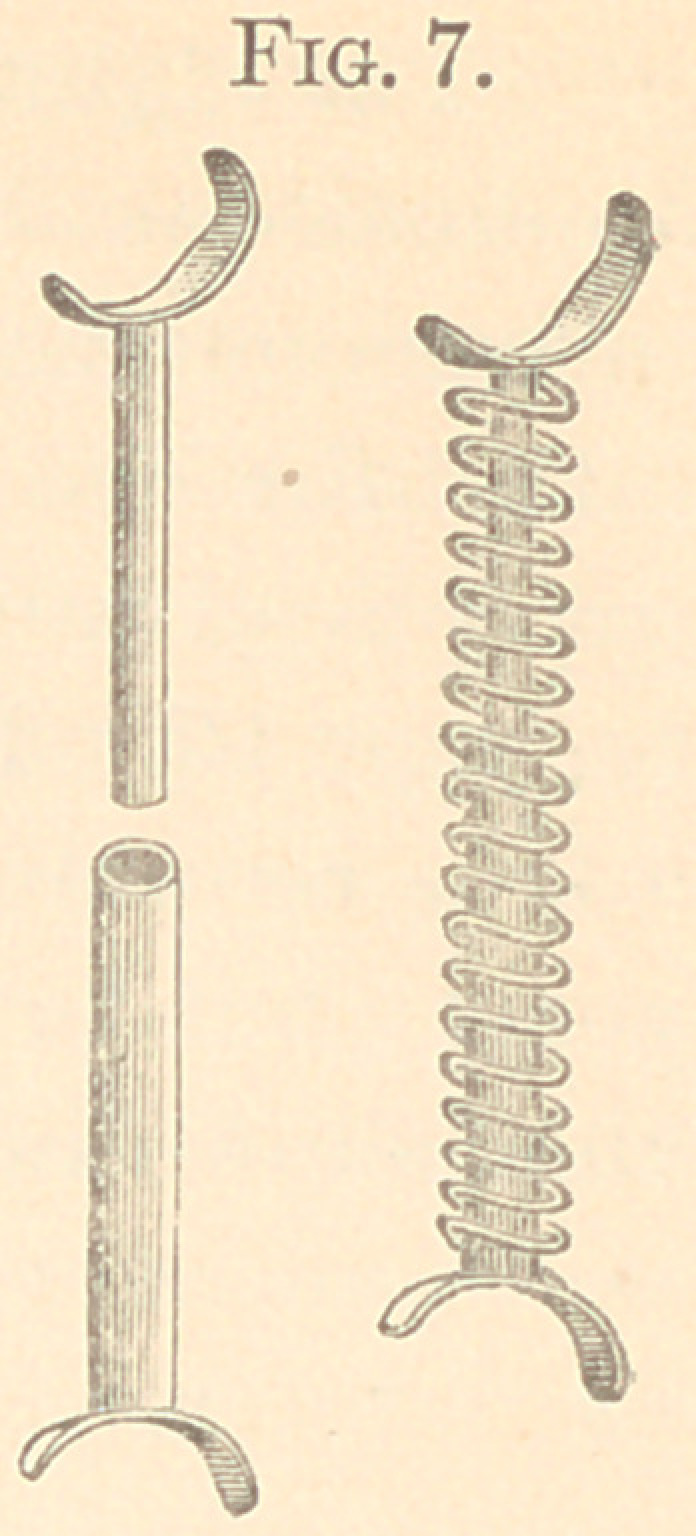


**Fig. 8. f8:**
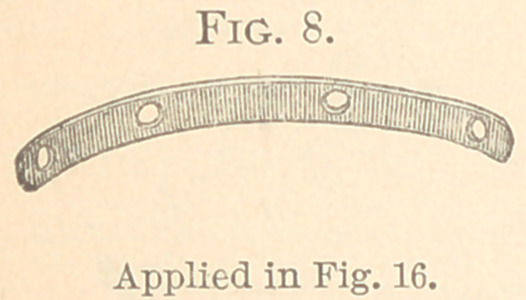


**Fig. 9. f9:**
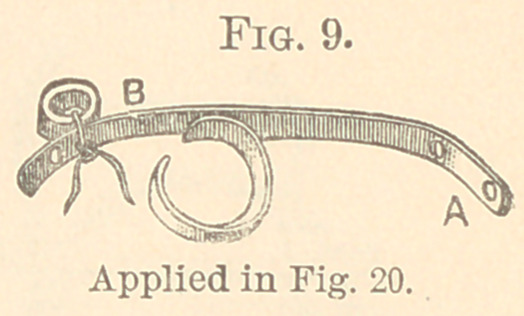


**Fig. 10. f10:**
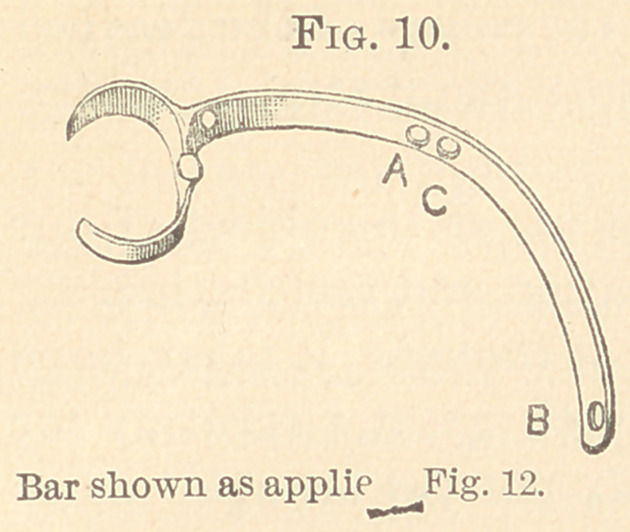


**Fig. 11. f11:**